# Cognitive Modeling of the Mnemonic Similarity Task predicts Alzheimer’s disease biomarker status in cognitively unimpaired older adults

**DOI:** 10.1002/alz.089582

**Published:** 2025-01-03

**Authors:** Casey R Vanderlip, Michael D Lee, Craig E.L. Stark

**Affiliations:** ^1^ University of California, Irvine, Irvine, CA USA; ^2^ University of California at Irvine, Irvine, CA USA; ^3^ The UC Irvine Institute for Memory Impairments and Neurological Disorders (UCI MIND), Irvine, CA USA

## Abstract

**Background:**

AD related pathologies, such as beta‐amyloid (Aβ) and phosphorylated tau (pTau), are evident decades before any noticeable decline in memory occurs. Identifying individuals during this asymptomatic phase is crucial for timely intervention. The Mnemonic Similarity Task (MST), a modified recognition memory task, is especially relevant for early AD screening, as it assesses hippocampal integrity, a region affected (both directly and indirectly) early in the progression of the disease. Further, strong inferences on the underlying cognitive mechanisms that support performance on this task can be made using Bayesian cognitive modeling. Here, we aimed to determine the efficacy of the MST, combined with cognitive modeling, at predicting AD biomarker status during this preclinical stage.

**Method:**

Using data from a prior study (Trelle et. al, 2021), 133 cognitively unimpaired older adults (age = 68.8±5.8, 63% female) were administered the MST and underwent a lumbar puncture to quantify AD biomarkers. All had a Clinical Dementia Rating of zero and normal neuropsychological test performance. Previously derived Aβ42, Aβ40, and p‐tau181 levels were used in the present analyses. Here, both traditional performance scores and cognitive modeling using multinomial processing trees was applied to each participants MST data using Bayesian approaches. We applied logistic regressions using both traditional metrics (“REC” and “LDI”) and model‐based metrics to predict Aβ and pTau status, assessing model efficacy via ROC analyses.

**Result:**

MST traditional metrics modestly predicted Aβ status (AUC = 0.64, p = 0.036). However, model‐based metrics were able to predict Aβ status at a higher level (AUC = 0.73, p = 0.020). We next assessed whether MST performance could predict pTau status. Our findings indicate that traditional metrics could not successfully predict pTau status (AUC = 0.50, p = 0.908). Conversely, model‐based metrics reliably predicted pTau status (AUC = 0.71, p = 0.037). Together, these results demonstrate that cognitive modeling of the MST can detect individuals with elevated AD biomarker status prior to cognitive decline.

**Conclusion:**

This work establishes the clinical utility of the MST as a screening tool for identifying older adults during the asymptomatic phase of AD which may reflect the optimal window for therapeutic intervention.

